# What is in an index? Construction method, data metric, and weighting scheme determine the outcome of composite social vulnerability indices in New York City

**DOI:** 10.1007/s10113-017-1273-7

**Published:** 2018-01-18

**Authors:** Diana Reckien

**Affiliations:** 1 Faculty of Geo-Information Science and Earth Observation (ITC), Department of Urban and Regional Planning and Geo-Information Management, University of Twente, Hengelosestraat 99, P.O. Box 217, Enschede, The Netherlands; 2 Earth Institute, Center for Research on Environmental Decisions, Columbia University in the City of New York, New York, NY USA

**Keywords:** Social vulnerability mapping, Index/indices construction, Variable addition/additive approach, Variable reduction approach, Principal component analysis (PCA), New York City

## Abstract

Mapping social vulnerability is a prominent way to identify regions in which the lack of capacity to cope with the impacts of weather extremes is nested in the social setting, aiding climate change adaptation for vulnerable residents, neighborhoods, or localities. Calculating social vulnerability usually involves the construction of a composite index, for which several construction methods have been suggested. However, thorough investigation of results across methods or applied weighting of vulnerability factors is largely missing. This study investigates the outcome of the variable addition—both with and without weighting of single vulnerability factors—and the variable reduction approach/model on social vulnerability indices calculated for New York City. Weighting is based on scientific assessment reports on climate change impacts in New York City. Additionally, the study calculates the outcome on social vulnerability when using either area-based (person/km^2^) or population-based (%) input data. The study reveals remarkable differences between indices particularly when using different methods but also when using different metrics as input data. The variable addition model has deductive advantages, whereas the variable reduction model is useful when the strength of factors of social vulnerability is unknown. The use of area-based data seems preferable to population-based data when differences are taken as a measure of credibility and quality. Results are important for all forms of vulnerability mapping using index construction techniques.

**Electronic supplementary material:**

The online version of this article (10.1007/s10113-017-1273-7) contains supplementary material, which is available to authorized users.

## Introduction

Assessing where socially vulnerable people live or work is assumed to be useful for urban adaptation policy and planning, guiding proactive, strategic climate change adaptation following the precautionary principle (Abson et al. 2012; Tran et al. 2009; Tran et al. 2010). Social vulnerability is considered a predisposition or precondition that makes some people or groups of people more susceptible to harm than others. Cutter and Finch (2008: 2301) define social vulnerability as “a measure of both the sensitivity of a population to natural hazards and its ability to respond to and recover from the impacts of hazards.” Similarly, in this study, social vulnerability is understood as a measure of sensitivity and the lack of adaptive capacity to a number of natural hazards—including those related to climate change—that may affect an individual, group, or community.

Social vulnerability studies integrate socio-economic aspects into the evaluation of climate impacts and coping capacity of individuals or households affected by climate shocks (Otto et al. 2017). This is important as socio-economic aspects can determine how a climate stressor affects a particular household. For example, vulnerability studies in Africa have shown that even minor climate perturbation can lead, e.g., to a famine when socio-economic and political conditions are unfavorable, whereas large climate shocks can be buffered if the contrary applies (Simelton et al. 2009). Socio-economic conditions can even determine the direction of influence of a climate event (positive or negative), with the well-off population sometimes being able to benefit during a period of hardship, e.g., by selling their stocks of food or hiring labor at low prices (Watts and Bohle 1993). Evidence points towards a number of implications for the less well-off households and those living in poverty, e.g., with regard to food security, displacement/migration, health, and safety (Otto et al. 2017). This shows that socio-economic aspects, as well as the cultural, institutional, and societal context are of importance for vulnerability studies.

Social vulnerability is commonly described by an index and mapped (Füssel 2007), often as composite index combining and aggregating multiple single indicators or sub-indices in various ways (Garschagen and Romero-Lankao 2013). The construction of indices can help to reduce complexity, to compare and rank results, and to communicate and present scientific outputs. Most of the approaches fall into two kinds—the variable addition and variable reduction approach. However, there are also less common approaches as summarized in Table 1.

**Table 1:** Overview of established construction methods for (social) vulnerability indices

	Name of approach/technique	Description	Methods used	Examples in the literature
1	Variable reduction approach or inductive approach	A large number of variables are used that potentially have an influence. These are reduced to the most influential components by merging variables that are highly correlated into a number of new variables or components. These are then normalized to a similar unit or variability; and then mapped	Factor analysis; principal component analysis (PCA)	Abson et al. 2012; Cutter et al. 2003; de Sherbinin and Bardy 2015; Schmidtlein et al. 2008; Yoon 2012; Holand et al. 2011; Tate 2012
2	Variable addition approach or deductive or additive normalization approach • With weighting • Without weighting	Only those variables are used that are very likely influential or those that have been determined as influential in previous studies. These variables are normalized, added, and mapped	Normalization of data via *z*-scores, min-max rescaling or similar and addition of scaled variables	Abson et al. 2012; Yoon 2012; Tate 2012
3	Sub-index approach or hierarchical approach	Here, first, a number of variables are identified that contribute to sub-indices similarly (added to equal shares to form 100% likelihood of a sub-index). Sub-indices are, e.g., sensitivity, coping, and adaptation. These are then added to get to the overall variable of vulnerability	Likelihood measures of susceptibility, coping, and adaptation are added to arrive at vulnerability	Welle et al. 2012; Tate 2012
4	Fuzzy normalization approach	Variables of importance for vulnerability are selected and joined via fuzzy reasoning, i.e., fuzzy membership functions for degrees such as “high” or “low” and the definition of respective threshold values	Addition of fuzzified variables	Lissner et al. 2011

Using quantitative indicators and their mapping is a common way of assessing social vulnerability to environmental and natural hazards (Tapsell et al. 2010). However, a number of challenges for the construction of vulnerability indices have been identified (Preston et al. 2011; Abson et al. 2012; Schmidtlein et al. 2008; Yoon 2012; Tate 2012). For example, large differences exist between estimations of vulnerability based on different component construction methods (Preston et al. 2011; Schmidtlein et al. 2008). Moreover, the use of different metrics, such as area-based (per km^2^) versus population-based (percent of inhabitants) variables produces divergent results (Eriksen and Kelly 2007). Scholars also see the difficulty of selecting (suitable) variables and the need to include more qualitative variables (Tapsell et al. 2010). Others criticize the widespread absence of weighting vulnerability factors (Brooks et al. 2005)—often due to a lack of robust criteria and replicable quantitative empirical evidence (Sullivan and Meigh 2005)—and the lack of verification (Eakin and Luers 2006; Eriksen and Kelly 2007). A number of these issues call for structural assessment (Hinkel 2011; Preston et al. 2011; Yoon 2012)—the main goal of this paper.

The main objectives of this paper are to compare social vulnerability indices across construction alternatives and metrics in New York City (NYC), United States of America (USA), investigating:

- The index change between weighted and non-weighted additive models
- The index change between population- vs area-based normalization of data
- The index change between additive and reductionist models

New York City lacks comprehensive social vulnerability studies. Recently, only one study on the social vulnerability to coastal flooding became available (de Sherbinin and Bardy 2015). This, however, only applies to the coastal areas using one method and one metric. Assessing the social vulnerability to environmental hazards over the entire urban area of NYC is an urgent research need. Doing so this study also aims to support the NYC planning legislature in climate change adaptation and social resilience building (New York City Press Office 2015).

### Case study: New York City

NYC serves as a perfect example with its complex urban environment, high densities, and stark social contrasts, potentially determining strong differences of social vulnerability within short distances. NYC is the most densely populated city of the USA with about 8.5 million inhabitants in the city (United States Census Bureau 2017d) and almost 20 million people in the conurbation in 2016 (United States Census Bureau 2017e). Manhattan (New York County) has a density of about 27,700 persons/km^2^ (United States Census Bureau 2017c).

New York has a rather evenly distributed ethnic and racial composition (33% white, non-Hispanic; 23% black or African American, non-Hispanic; 13% Asian; 29% Hispanic or Latino; < 1% Native American) (United States Census Bureau 2017d). However, among boroughs, there are large differences in ethnic/racial composition, amenities and income. NYC has the third highest rate of income inequality among US cities: the lowest paid 20% of residents earn on average US$12,300 and the highest paid 20% US$ 489,000 (Long 2014).

The climate is temperate, semi-humid, and maritime (Lauer and Frankenberg 1992) with cool and damp winters and hot and humid summers. The annual precipitation of about 1200 mm (1910–2013) is spread relatively evenly across the year (The Weissman Center for International Business 2015). Hurricanes—large storm systems that bring high-speed winds and normally lots of rain from the Gulf of Mexico—can affect the city in summer/autumn (the hurricane season lasts from 1 June to 30 November). Nor’easters—North-Easterly winds that are usually accompanied by large amounts of rainfall from the North East Atlantic Ocean—can affect the city in winter. The city is also prone to heat waves, usually accompanied by high humidity (Rosenzweig and Solecki 2010) as NYC is located close to the sea. Four of NYC’s five boroughs are an island or part of an island.

Climate projections for NYC suggest that heat waves (three or more consecutive days with maximum temperature exceeding 90 °F (~ 32 °C)) will approximately triple in frequency by the end of the century compared to current conditions (Horton et al. 2011). In 2050, NYC is projected to have a climate similar to that of present-day St. Louis (Kalkstein and Greene 1997). Knowlton et al. (2007) estimate an increase of 47–95%—with a mean of 70%—in excess heat-related deaths for the NYC area from 1990 to 2050. Precipitation is expected to decrease overall for the north-eastern region of the USA (Blake et al. 2000). However, seasonal increases in winter precipitation may put a burden on areas that are already exposed to flooding and other rain-related hazards (Horton et al. 2010).

## Materials and methods

### Data

Cutter et al. (2003) serve as the basis for indicator selection, being a milestone in the development of quantitative approaches of social vulnerability assessment. Their comparative study of US counties uses over 250 indicators derived from the literature. The results highlight the importance of income, age, building density, quality of housing stock, ethnicity/race, economic and infrastructure dependency as well as service orientation for the social vulnerability to natural hazards. Using 42 final independent, normalized variables in a principal component analysis (PCA), the study yielded 11 components^1^ of vulnerability. This study laid the groundwork for a uniform approach to measuring social vulnerability across space and time (Schmidtlein et al. 2008) and the method has been applied repeatedly to other areas (Abson et al. 2012; Rygel et al. 2006; Tate 2012).

Many indicators that Cutter et al. (2003) used are now well-established and researched. For example, old and young age are potent social vulnerability factors (Curriero et al. 2002; Luber and McGeehin 2008; McGeehin and Mirabelli 2001; Reckien et al. 2017), which are, considering the demographic shift of industrialized societies and their age structures in cities, very important for urban areas (Curriero et al. 2002). Poverty and low income as well as poor health have been shown to increase sensitivity in many cities and countries (Luber and McGeehin 2008; McGeehin and Mirabelli 2001; Nair et al. 2013), while factors such as the availability of personal air conditioning during heat waves (Luber and McGeehin 2008) and the degree of integration into the community or neighborhood (Klinenberg 2002, 2013) are shown to lower excess mortality during heat. Factors that contribute to social vulnerability might differ depending on the stressor, such as heat waves, floods, or storm surges, but the list of Cutter et al. (2003) covers a wide range of factors from census data that have found to be important in many studies and for many stressors (Tapsell et al. 2010).

Some indicators originally proposed by Cutter et al. (2003) are not relevant in NYC, such as percentages of people employed in extractive industries, of housing units that are mobile homes, and of Native Americans. Those indicators were not considered. Built environment indicators were also omitted to focus more explicitly on individual social characteristics, as suggested by Borden et al. (2007) and Schmidtlein et al. (2008). Cars/household was added as this was found important in NYC, based on the review of planning documents (Table 2).

**Table 2:** Results of text analysis: number of hits per keyword (i.e., salient term related to social vulnerability) and average number of hits per page in New York City‘s climate change impact assessment reports

Keyword plan	Justice	Poverty	Elderly	Kids	Ethnicity	Gender	Cars	Total hits	Total pages	Average hits per page
1. Metro EC	4	2	1		3			10	24	0.4
2. CC Ass + Action								–	102	–
3. NYC Nat Hazards		14	18	8		2		42	471	0.09
4. CC & Adap in NYC	2	4	1					7	349	0.02
5. NYS SLR TF	6	4	2	1	2			15	103	0.15
6. Vision 2020	1	6		7				14	192	0.07
7. PlaNYC 2011	3	16	8	15				42	202	0.21
8. NYS ClimAID	3	21	13	4	9	1	1	52	57	0.91
Total hits	19	67	43	35	14	3	1	182		

Column 1 shows themes of summarized keywords (see Supplementary Methods). Abbreviated titles of planning documents are as follows: 1, Climate Change and a Global City: an Assessment of the Metropolitan East Coast Region, Assessment Synthesis; 2, Climate Change Program Assessment and Action Plan, Report 1; 3, New York City Natural Hazard Mitigation Plan; 4, Climate Change and Adaptation in New York City: Building a Risk Management Response; 5, New York State Sea Level Rise Task Force—Report to the Legislature; 6, Vision 2020: New York City Comprehensive Waterfront Plan; 7, PlaNYC Update April 2011—a Greener, Greater New York; 8, Responding to Climate Change in New York State—ClimAID Synthesis Report

Following these reflections, the following indicators are used:

1. Total population [km^2^]; 1
2. Female population [km^2^, %]; 1
3. Population of black people or African American (one race) [km^2^, %]; 1
4. Population of Asian people (one race) [km^2^, %]; 1
5. Population of Hispanic people [km^2^, %]; 1
6. Population of children < 10 years of age [km^2^, %]; 1
7. Population of people aged 65 and higher [km^2^, %]; 1
8. Population living in poverty [km^2^, %]; 1
9. People without access to a car [km^2^, %]; 2
10. One-person households [km^2^, %]; 1

The data was obtained from the 2010 US Decennial Census (1) and the 2006–2010 American Community Survey (2) through American Fact Finder (United States Census Bureau 2017a). The study relies on 2010 data—the last official census—assuming that the socio-economic situation in the city is still roughly the same.

The spatial level is census tracts (United States Census Bureau 2017b) covering Bronx County (the Bronx), Kings County (Brooklyn), New York County (Manhattan), Queens County (Queens), and Richmond County (Staten Island). All data were processed using the US Census guideline (United States Census Bureau 2017f). Data for each tract were processed in two ways: as person per km^2^ (area-based data) and as percentage of total residents per tract (population-based data). Uninhabited tracts were deleted (e.g., those for parks, industrial areas, or others without housing units).

Additional data on area and point landmarks, water areas and lines, census tract, and county borders were retrieved from the Census Bureau using Tiger file shapes.

### Methods

#### Additive normalization approach with and without weighting

The additive normalization approach is based on the summation of values of multiple indicators that are assumed to strongly contribute to social vulnerability in the area and subject of interest, after these indicators have been normalized to a unitless, comparable scale (Abson et al. 2012; Tran et al. 2009). Normalization of indicators provides a linear transformation that preserves the ranking and correlation structure of the original data and allows for indicators with different scales to be summed (Tran et al. 2010).

Indicators were rescaled to values between 0 and 1 using maximum value transformation (Yoon 2012) and then summed with or without weighting. The final sums can be normalized again to yield an index in the range of 0 to 1, for direct comparison with the PCA-based vulnerability indices (Abson et al. 2012).

##### Identification of a weighting procedure: New York City’s climate change impact assessment reports

The weighting of indicators is based on a review of NYC planning documents and assessment reports that deal with natural hazards, environmental change and/or climatic change. It is thereby an innovative and methodologically defensible model for weighting and a triangulation of location-specific social vulnerability indicators.

For this study, eight plans were selected that target climate change impacts, adaptation, or resilience (Table 2). These documents are based on scientific analysis and/or expert judgment, produced by scientists and policy makers working in the NYC area (Solecki 2012). Plans that exclusively target climate change mitigation are omitted, as are those that are solely carried out by research institutions without a connection to the city’s planning boards. Reports that address single sectors or sub-goals of the PlaNYC—the master planning framework drawn up by Mayor Bloomberg in 2009 to tackle NYC’s economic, infrastructural and ecological problems, and studies—are also neglected.

The analysis of the assessment reports includes text analysis using social vulnerability salient “keywords,” which are provided as Supplementary Methods. Combined with a context analysis, mismatches could be avoided and the relevance for social vulnerability to environmental change and natural hazards be confirmed. Table 2 provides an overview of the reviewed documents and shows the results of the text analysis. Supplementary Text S1 and Supplementary Table S2, S3, and S4 provide the full analysis.

Table 2 reveals that the majority of NYC’s climate change impact assessment reports until the year 2014 does not focus on social vulnerability or equity issues, nor does the master planning framework PlaNYC. The focus was on the protection of the waterfront from storm surges, hurricanes, and sea level rise (City of New York and Department of City Planning 2011; Solecki 2012). Less attention had been paid to heat wave risk. There is only one detailed and comprehensive study of social vulnerability in NYC—the report “Responding to Climate Change in New York State” (ClimAID). As this is the only report with a true focus on social issues, that addresses ethnicity and race as well as gender, and shows hits for all categories of vulnerability indicators—with a similar distribution of hits as compared to the average of all reports (except for the keyword “kids,” which was a focus of PlaNYC)—the ClimAID study serves as the basis for weighting the vulnerability factors, accomplished as follows: Each vulnerability factor was weighted by the respective number of hits in the ClimAID report, i.e., female population, population without cars, and single households by 1; population of African American, Asians, and Hispanics by 9; population of children by 4; population of elderly by 13; and population of households living in poverty by 21.

#### Variable reduction approach

The variable reduction approach is based on the use of many, potentially all available indicators of social vulnerability in the study area and for the subject of interest. It reduces partly collinear indicators to a smaller number of unitless, uncorrelated components using PCA (Eriksen and Kelly 2007; Abson et al. 2012). The work of Cutter et al. (2003) serves as a blueprint. The manual, the so-called SoVI recipe, is a detailed description of the underlying methodology (University of South Carolina 2011). It was applied on all ten indicators listed above.

This includes performing a PCA using varimax rotation, selecting components with an eigenvalue superior to one (Kaiser Criterion). Varimax rotation tends to load each variable highly on just one component easing component interpretation (Schmidtlein, et al. 2008). Subsequently, principal components were adjusted for cardinality, i.e., inverted by multiplying the scores by − 1, so that components have the same directional influence on social vulnerability, i.e., all increase or all decrease social vulnerability. Finally, the component values were summed with equal weights and normalized again (min-max normalization) to arrive at values between 0 and 1, allowing better comparison with the results of the additive approach (Yoon 2012).

## Results

### Vulnerability changes in weighted and non-weighted additive models

#### Additive models

**Fig. 1:**
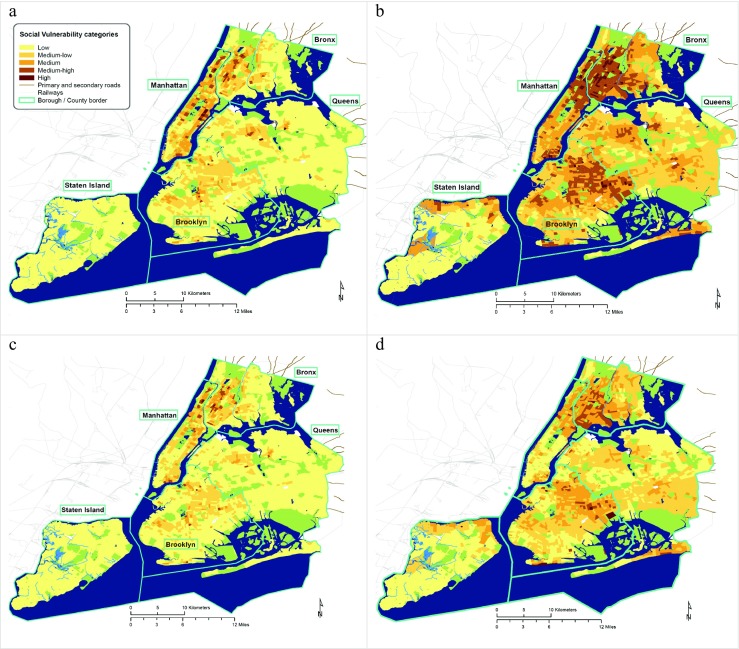
Social vulnerability indices for New York City calculated using the additive model with and without weighting, and different data metrics. **a** The outcome of the additive model without weighting based on area-based data (person/km^2^). **b** The outcome of the additive model without weighting based on population-based data (%). **c** The outcome of the additive model with weighting based on area-based data (person/km^2^). **d** The outcome of the additive model with weighting based on population-based data (%). Non-residential areas are shown in blue (water bodies), green (parks), and white (industrial areas, etc.)

Figure 1 reveals that the patterns of vulnerability across the models are largely the same, although there are differences in the extent or intensity of the index. The most vulnerable areas are to be found in the Bronx, Uptown Manhattan, the two Bridges Neighborhood in the Lower East Side of Manhattan, Central and Eastern Brooklyn, Central and Western Queens, as well as Northern Staten Island.

The review of NYC planning documents (Table 2) shows that “poverty” is the keyword with most hits across all reports; it occurs in about 50% of the plans. The “elderly” and “children” are the second and the third most frequently mentioned keywords, respectively. Poverty can therefore be taken to be the most important factor contributing to social vulnerability in NYC, followed by old age. Therefore, the weighted approach increases vulnerability in areas of residents living in poverty, of high age, and also ethnic minorities. The unweighted approach may overestimate vulnerability in well-off and predominantly white neighborhoods.

To elicit the influence of weighted versus non-weighted factors in the additive model I compare the “additive points”—the sum of vulnerability values for all tracts, as well as the “absolute change”—the sum of the absolute differences in vulnerability values between models, metrics, or weighting options. Table 3 shows that the unweighted and weighted index produce differences in the extent or severity of social vulnerability (additive points). The additive model without weighting classifies larger areas as highly vulnerable (higher values under “additive points”), as compared with the additive model with weighting. Applying a weighting stretches the range of the index, thereby reducing the number of cases in the highly vulnerable category.

**Table 3:** Comparison of social vulnerability indicators between models when normalized to 0 and 1. Additive points are the sum of all cells’ vulnerability values. Absolute change is the sum of the absolute difference of this vulnerability value between models

		Additive model w/o weighting	Additive model with weighting	PCA model	Difference (with and without weighting)	Difference (additive w/o weighting and PCA)	Difference (additive with weighting and PCA)
Area-based data (person/km^2^)	Additive points	469.03	394.20	339.88	74.83	129.15	54.32
Population-based data (%)	Additive points	984.87	670.60	560.22	314.27	424.65	110.38
Difference (person/km^2^ and %)	Absolute change	515.84	276.40	220.24			

Comparing the weighted and unweighted model in its spatial outcome (Supplementary Figure S1), we see that the additive model with weighting shows higher vulnerability values for tracts where people are living in poverty. For the area-based data this is the case in the Central Bronx, parts of Central Staten Island, as well as patches in Queens and Brooklyn. In these areas, the density of socially vulnerable residents is high. In the population-based map mainly Staten Island comes up, showing a high vulnerability of people living in poverty percentage-wise.

### Vulnerability changes in population- vs area-based normalization of data

#### Additive models

Table 3 also shows that there are substantial differences between vulnerability scores when the input data is based on different metrics, i.e., person/km^2^ and %. The difference is highest for the additive model without weighting (522.87 of absolute change as compared to 327.02).

Looking at the differences between the two additive models, Table 3 shows that the area-based input data produces smaller differences as compared with the population-based input data (88.03 absolute change as compared with 319.94). This is again due to the spread of the data. The area data has a larger spread/variability than the population data (see Supplementary Figure S2 for histograms and statistics); hence, area-based data reduces the number of extreme cases and produces lower values and lower absolute change, respectively.

The large differences between area-based and population-based data cause some differences across space, although the patterns of vulnerability remain largely similar. At times single tracts can change from a low category in the area-based calculations to a medium or medium-high vulnerability category in the population-based calculations (Fig. 1). When the index is higher using area-based data, one can expect a higher density of vulnerable people as compared with the vulnerability index of percentage-based data. This is the case in large parts of Manhattan—the Upper East Side, Upper West Side, and Midtown to the Lower East Side (Supplementary Figure 3). In these neighborhoods, the social vulnerability is relatively low percentage-wise, but somewhat higher area-wise (referring to high population density).

#### Reductionist models

Table 4 shows the results of the PCA analysis, which yielded three principal components (PC) using both area- and population-based data. In the area-based model, the PCA singles out elderly households without cars, poor Hispanic families, and Asian households, which explain about 87% of the variance. In the population-based model, we find Hispanic families and lone elderly, high density areas without cars, and African-American and female households. That model explains 64% of the variance in the data. Based on the explained variance, one would favor the area-based model. Content-wise, the components differ, although they have similar principal variables. Hispanic families, the elderly and kids, and female, carless, and single households are strongly influential in both models.

**Table 4:** Results of principal component analysis, with data description and sources

	PC	Name	Variance explained	Principal variables	Correlation
Area-based data (person/km^2^)	1	Elderly HH w/o car	60.48	• Total population • Female population • Population of people aged 65+ • People without access to a car • One-person households	0.725 0.749 0.855 0.888 0.965
	2	Poor Hispanic families	16.27	• Total population • Female population • Hispanic population • Population of children < 10 years • Population living in poverty	0.663 0.641 0.877 0.874 0.899
	3	Asian HH	10.16	• African Americans (one race) • Asian population (one race)	− 0.669 0.858
		Total	86.91		
Population-based data (%)	1	Hispanic families and lone elderly	27.70	• Hispanic population • Population of children < 10 years • Population of people aged 65+ • One-person households	0.628 0.796 0.669 − 0.688
	2	Dense areas w/o cars	19.83	• Total population • People without access to a car	0.794 0.922
	3	African American and female HH	16.50	• Female population • African Americans (one race) • Asian population (one race)	0.682 0.817 − 0.694
		Total	64.03		

*HH* households

**Fig. 2:**
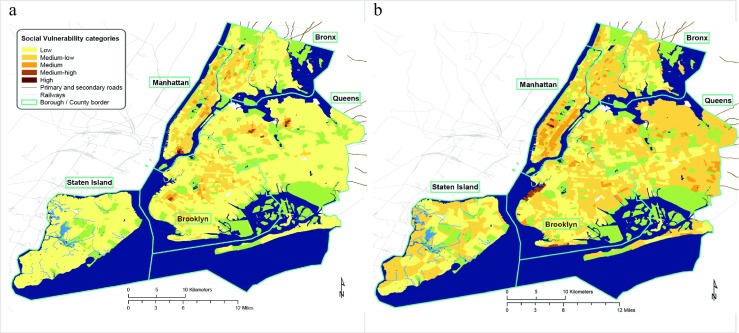
Social vulnerability indices for New York City calculated using principal component analysis and different metrics. **a** The outcome based on area-based data (person/km^2^). **b** The outcome based on population-based data (%). Non-residential areas are shown in blue (water bodies), green (parks), and white (industrial areas, etc.)

The quantitative differences of the calculated vulnerability using PCA and different metrics are shown in Table 3 and Fig. 2.

Figure 2 and Table 3 show that compared to population-based data indices on area-based data classify less of the urban area as highly vulnerable. This is due to larger differences in the data per km^2^, which in turn leads to more tracts being classified as low and medium vulnerability. This could also be one reason why the area-based data produces more patches of high social vulnerability.

The pattern of vulnerability in both maps differs. In the area-based map, patches of high social vulnerability are found in the Two Bridges Neighborhood of the Lower East Side of Manhattan, in Borough Park/Brooklyn, as well as the Corona neighborhood and Flushing/Queens. For the PCA using population-based data, patches of high social vulnerability are to be found in Midtown Manhattan and Sunset Park/Brooklyn.

The changes are also documented in the Supplementary Figure S4, showing that area-based data produces higher vulnerability scores in the inner city areas. This is to be expected as these areas have higher population densities. In contrast, population-based data level out differences between areas of high and low population densities.

### Vulnerability changes between additive and reductionist models

The statistics (Table 3) and maps (Figs. 1 and 2; Supplementary Figure S5) show that there are profound differences between the additive and the reductionist model. The reductionist model misses the stress of social vulnerability in areas such as the Central Brooklyn, Upper Manhattan, and the South and Central Bronx—areas which would classically be perceived as housing socially vulnerable communities. Particularly when using population-based data, the reductionist model emphasizes parts of town that would not necessarily be perceived as socially vulnerable, such as Midtown Manhattan and some parts at the administrative fringes of the city.

**Fig. 3:**
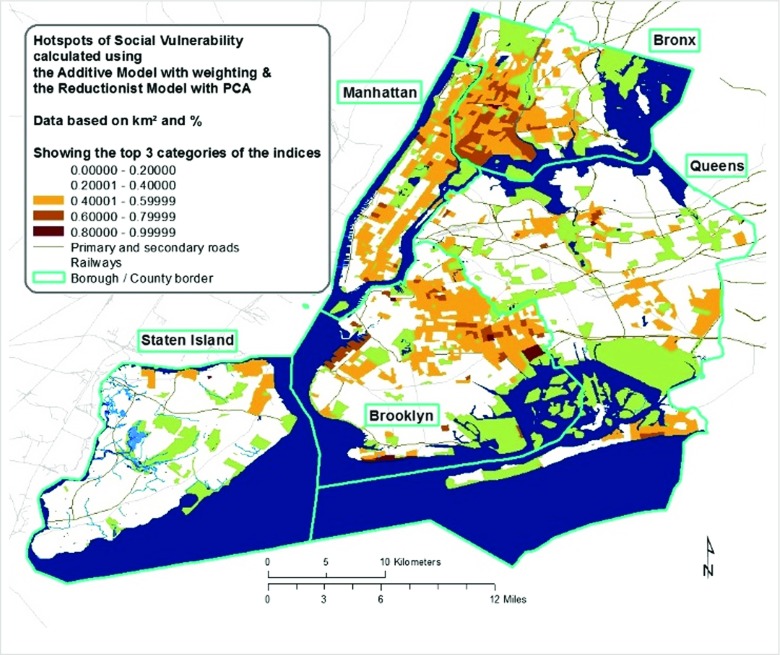
Hotspots of social vulnerability in New York City, based on different models and both area-based and population-based input data. Non-residential areas are shown in blue (water bodies), green (parks), and white (industrial areas, etc.)

It is difficult to judge which of the maps is a more realistic account of the actual or the perceived social vulnerability of NYC’s residents without comparing the indices with real-world damage or damage perception indicators for different climate stressor. Based on this analysis, I cannot judge the quality of indicators. To account for this uncertainty and to cater for the aim to support the NYC planning legislature in climate change adaptation and social resilience building, Fig. 3 shows an overlay of the patches of high social vulnerability, termed social vulnerability hotspots, as produced by both models and both types of input data.

## Discussion

This study investigated the changes of social vulnerability indices to natural hazards across construction methods and data metrics in NYC. It aimed to contribute to progress in indicator studies regarding social vulnerability to environmental change and natural hazards. The study finds large differences between approaches and metrics. Identified disparities are particularly large between construction methods, i.e., the additive and reductionist approach of indicator construction, although the results also highly differ between metrics, i.e., population- and area-based input data. Differences between construction methods and input data are in line with other studies showing disparities between (other) methodological aspects of indicator construction (Abson et al. 2012; Tate 2012).

The literature shows that most scholars use the reductionist approach with factor analysis or PCA. This may be due to the easiness of data selection, i.e., one can use all available data that is potentially related to vulnerability in the study area and does not need to care about their role, level of contribution, or correlation of single factors. As Abson et al. (2012) note, a variable reduction approach is used when it is assumed that correlating indicators are compatible and interchangeable and favored when variables strongly interact with each other or interact in complicated ways that cannot be captured through a simple arithmetic combination of individual factors. However, the reductionist approach is not favored when the contribution of individual indicators to an overall social vulnerability score is known to be high. The use of PCA will most likely underestimate their importance as indicators will be treated equally when using PCA—although components can be weighted, but more often are not (Cutter et al. 2003). If a weighting is applied components are often multiplied by the variance they explain, such as in de Sherbinin and Bardy (2015)—and not necessarily by their contribution to social vulnerability. Overall, the reductionist approach cannot rule social vulnerability contributions of single factors. Another disadvantage of the reductionist approach is the difficulty of interpretation and therefore of relation to real-world decision-making. As factors are merged into components on the basis of statistical, rather correlational and not content-driven reasoning, the final PCA-based social vulnerability index is difficult to communicate to stakeholders. One can map the principal factor of a component as a driver, but doing so does not add any information as compared to mapping all influential vulnerability factors right in the beginning (without conducting the PCA). For that reason, using the reductionist approach seems to lack application potential.

The additive approach is favorable when it is assumed that each vulnerability factor adds a different element of vulnerability to the overall composite index (Abson et al. 2012). Adding up differential contributions is not unreasonable, as vulnerability factors can be additive. For example, living in poverty is assumed to represent a form of vulnerability (people do not have financial means to remedy in a hazardous situation), while belonging to a social/racial minority is another (for example, due to restricted access to media and different sources of information, language barriers). Likewise, elderly people may be sensitive due to poor health and also because they do not own a car (affecting the ability to flee, i.e., drive away). All these factors may be related, but they relate to different underlying causes of vulnerability, which become important in different extreme weather situations. Single factors can be weighted to account for their influence and strength on the composite index, although most authors refrain from weighting due to the lack of an appropriate basis for weighting. The weighting in this study is based on a detailed and comprehensive study of social vulnerability in NYC—the report “Responding to Climate Change in New York State” (ClimAID). Additionally, the additive approach does not distort influences of single factors in the overall index. It is therefore easier to comprehend and to communicate to stakeholders, which increases the application potential. I have made the experience that stakeholders favor the additive approach.

Overall, Abson et al. (2012) support using PCA, as to their view, it involves fewer trade-offs between communicability and the information richness than the additive approach. They regard the additive approach as (more) problematic (Abson et al. 2012), since the underlying causes of vulnerability are lost in the final summative index, i.e., locations may yield the same degree of vulnerability due to different reasons—whereas different drivers of vulnerability (even if weirdly merged) are clearly distinguished in-between the components using PCA. However, these problems can be overcome by consulting maps of individual indicators. And, consulting maps of individual factors or components is also necessary for revealing causes of social vulnerability calculated via PCA. Therefore, others have questioned the credibility of the PCA model (Tate 2012).

This study sees more potential in the additive approach, not only because of the deficiencies in relating to stakeholders. Another aspect of confusion in a PCA analysis regards the number of input data. Here, ten input variables yielded three components and covered up to 87% of the variance of the (area-based) data. In another study, using 20 input variables with five components (de Sherbinin and Bardy 2015), 73% of the variance of the (population-based) input data was covered. I can therefore conclude that using more input variables does not necessarily lead to a larger explained variance. The latter point also lends to the use of area-based data.

Using different metrics produces differences in social vulnerability that are profound, as found by others (Tate 2012). And, area-based input data does not only explain more of the variance in the PCA but also produce lower differences across models. The use of area-based data seems advantageous considering (1) the density being a potent vulnerability factor (Cutter et al. 2003), (2) the concordance of results based on area-based data with the vulnerability assessments in the policy and planning literature, and (3) the robustness of results (i.e., relatively small differences between models). Thus, it seems that the relative infrequent use of area-based data may be unwarranted.

However, a critical question is whether it is more important that densely populated areas are more vulnerable (more people are affected at once) or emergency responses can be more effective (more people can be helped at the same time). Dense areas also bear the chance of interaction among community members, which can be decisive. Although community interactions seems more influenced by cultural aspects than by density (Klinenberg 2002). Overall, this work underlines the severity of the choice of construction models and particularly the choice of metrics when using indicator studies (Tate 2012).

Regarding the findings, this study shows that NYC’s policy and planning documents with reference to climate change impacts, adaptation, resilience, and environmental hazards identify low income residents, the elderly, and ethnic minorities (African American, Hispanic, and Asian residents), in this order, as being particularly vulnerable to environmental hazards. This is in line with other studies of social vulnerability for other US cities and counties, e.g., Tate (2012) for Norfolk/Virginia, Sarasota Count/Florida, Nueces/Texas, and Klinenberg (2002) for Chicago.

Also spatially, this study determined to a large extent similar areas as socially vulnerable as, e.g., found in de Sherbinin and Bardy (2015) study of social vulnerability to coastal flooding. Their results of the reductionist approach with 20 input variables and five components show similar vulnerable areas as revealed in the additive approach without weighting (population-based input data) and PCA (population- and area-based input data). I therefore conclude that the use of the reductionist approach with PCA using more input variables and more components, first, produces spatially similar results to the output of the additive approach without weighting (population-based data) and, second, eases out the differences between population- and area-based input data. From this follows that the reductionist approach is indeed best be employed with large input data, and that in this case, potentially, the differences between metrics (population- or area-based input data) become less important.

Unfortunately, though, this study does not offer progress in comparing statistically constructed social vulnerability indices with the actual degree of impact, affectedness, damage, harm, or burden experienced during climate-related events—an urgent research need (Eriksen and Kelly 2007; Fankhauser et al. 1999; Füssel 2007; Hinkel 2011; Preston et al. 2011; Schmidtlein et al. 2008; Tran et al. 2009). Without a comparison of statistical vulnerability measures and real-world burden indicators, the usefulness and credibility of social vulnerability mapping must be questioned—in particular because of the large differences found. de Sherbinin and Bardy (2015) offer some insights in that respect, showing that—for NYC—socially vulnerable areas were not differently impacted by hurricane Sandy’s flood extent than other less vulnerable areas. Similarly, other studies suggest that the relation between, for example, flood exposure after Hurricane Sandy and mental health outcomes is complex (Lieberman-Cribbin et al. 2017; Mongin et al. 2017); concordance between statistical indicators and real-world impacts exists only partially. Further studies are needed to investigate the conformity between real-world damage and statistically constructed indices, hence the usefulness of constructing socially vulnerability indices. And, again, all statistical approaches presume the availability of data. Non-measureable, more qualitative factors of vulnerability or resilience, e.g., functioning social support networks, cannot be captured with either approach. Integrating more qualitative measures may be required in order to be able to move from mapping social vulnerability to analyzing the dynamics of social resilience (Eakin and Luers 2006).

## Conclusion

The results show that both construction methods and metrics of input data are influencing the outcome of indicator-based social vulnerability studies. The metric is very important for both the additive and the reductionist models. Using the differences between metrics and models as a measure of robustness, and robustness as an indicator of quality, I conclude that the use of area-based input data (person/km^2^) is favorable to population-based (%) data.

Regarding the construction methods, applying a weighting increases the spread of data and therefore reduces the height of vulnerability indices, that is, less areas classify as highly vulnerable. Moreover, weighting highlights crucial vulnerability factors, which in the additive model relate to poverty and the elderly and are in NYC mostly located in the Bronx, Northern Manhattan, Central East Brooklyn, and Central West Queens. Overall, area-based input data stresses vulnerability due to density; population-based input data stresses social vulnerability percentage-wise.

The documented results show that it is crucial to understand the implications of using different construction methods and metrics of input data, as these are substantially influencing the outcome of (social) vulnerability indices. The documented results are important for all forms of vulnerability mapping using index construction techniques.

## Electronic supplementary material

ESM 1 (DOCX 1914 kb)

## Footnotes

1 The 11 components comprise: (1) personal wealth (per capita income), (2) age (median age), (3) density of the built environment (no. of commercial establishments per square mile), (4) single-sector economic dependence (% employed in extractive industries), (5) housing stock and tenancy (% housing units that are mobile homes), (6) race (African American) (% African American), (7) ethnicity (Hispanic) (% Hispanic), (8) ethnicity (Native American) (% Native American), (9) race (Asian) (% Asian), (10) occupation (% employed in service occupations), and (11) infrastructure dependence (% employed in transportation, communication, and public utilities).
