# Supplementary material for: What is in an index? Construction method, data metric, and weighting scheme determine the outcome of composite social vulnerability indices in New York City
Source: Reg Environ Change. 2018 Jan 18;18(5):1439–51. doi: 10.1007/s10113-017-1273-7 (PMC6448355; doi:10.1007/s10113-017-1273-7)
Supplement: Supplementary file 1 — (DOCX 1914 kb) [file 10113_2017_1273_MOESM1_ESM.docx]

**Supplementary Material**

**What’s in an index?**

**Construction method, data metric and weighting scheme determine the outcome of composite social vulnerability indices**

**in New York City**

**Diana Reckien**

**Current affiliation**: University of Twente, Faculty of Geo-Information Science and Earth Observation (ITC), Department of Urban and Regional Planning and Geo-Information Management, Enschede, The Netherlands

**Affiliation where this work was carried out**: Columbia University in the City of New York, Earth Institute, Center for Research on Environmental Decisions, New York, USA

**Address**: 1) P.O. Box 217, 7500 AE Enschede, The Netherlands;

2) Hengelosestraat 99, 7514 AE Enschede, The Netherlands

**E-mail**: [d.reckien@utwente.nl](mailto:d.reckien@utwente.nl); [dianareckien@gmail.com](mailto:dianareckien@gmail.com)

**Tel.:** +31 53 4874 525

Contents

[Supplementary Methods 2](#_Toc487284621)

[Supplementary Text S1 - Short summaries of climate-related planning documents for New York City 3](#_Toc487284622)

[Supplementary Table S2 - List of planning documents analyzed in NYC 5](#_Toc487284623)

[Supplementary Table S3 - Planning practice: Are NYC’s planning strategies socially integrative? (Planning document study) 6](#_Toc487284624)

[Supplementary Table S4 – Detailed overview of hits per hit category and report 14](#_Toc487284625)

[Supplementary Table S5 - Comparison of Social Vulnerability Indicator when using the additive model with and without weighting and the reductionist model with PCA 15](#_Toc487284626)

[Supplementary Figures S1 16](#_Toc487284627)

[Supplementary Figures S2 – Absolute change between models with and without weighting 17](#_Toc487284628)

[Supplementary Figures S3 – Histograms and statistics 18](#_Toc487284629)

[Supplementary Figures S4 – Absolute change between metrics 1 19](#_Toc487284630)

[Supplementary Figures S5 – Absolute change between metrics 2 20](#_Toc487284631)

## Supplementary Methods

The literature content analysis involved searching for following keywords:

- inequality, inequity, equity, justice
- low-income, income [including ‘mixed income’]
- poverty, poor [in relation to people]
- homeless
- elderly/elderlies, senior/s, old
- children, kids, infant
- race, racial, ethnicity, ethnic
- minority, minorities
- African American, African-American, black [in relation to people], color [in relation to people]
- Hispanic, Latino
- gender, sex, female, women

## Supplementary Text S1 - Short summaries of climate-related planning documents for New York City

The awareness for social vulnerability of place to climate and weather extreme events in NYC started with the *Metro East Coast Study* (Metro EC) in 2000. The report repeatedly stresses the inequalities of income, minorities and races as a current and increasing challenge to NYC’s future. However, the report neither details out differences across sensitivity factors, nor refers to climate change or weather extremes in particular. Also, it does not state proposals for action.

The *Climate Change Program Assessment and Action Plan* (CC Ass + Action) (2008) of the NYC Department on Environmental Protection lacks any socio-economic salient terms. The assessment plan addresses climate science, impacts of climate change, adaptation options, and mitigation efforts and out of a total of six chapters three focus on impacts and/or adaptation. The plan concentrates on first-order impacts, i.e. impacts on the infrastructures, than on social aspects.

The *Natural Hazard Mitigation Plan* (NYC Nat Hazards) (2009) of the NYC Office of Emergency Management, does address social vulnerability. Various social vulnerability factors are mentioned and mapped across the NYC area in order to locate places of social vulnerability as part of NYC’s ‘hazard environment’, as they call it. A number of recommendation target sensitive populations, particularly low-income seniors. Impacts are expected during extreme heat and cold. The plan refrains from associating sensitivity to ethnic minorities and people of color.

The volume of the *Annals of the NYC Academy of Sciences ‘Climate Change and Adaptation in New York City: Building a Risk Management Response’* (CC & Adap in NYC) of includes few social vulnerability related terms and only one recommendation targeting social vulnerability, i.e. low-income home-owners. In general this volume is more directed towards impacts on and adaptation of NYC’s infrastructures.

The *New York State Sea Level Rise Task Force - Report to the Legislature* (NYS SLR TF) refers to social vulnerability at various places in the report and dedicates a whole chapter to climate justice. Specific recommendations are given to include low-income communities of color into the resiliency planning process.

New York City’s *Vision 2020 - Comprehensive Waterfront Plan* (Vision 2020) speaks in favor of mixed-income opportunities and housing and is not particularly targeted towards social sensitive groups. The report focuses on what the waterfronts can provide and how it can be developed. The climate resilience chapter outlines impacts and strategies, but does not refer to social vulnerability.

*PlaNYC–A greener, greater New York* (PlaNYC 2011) is probably the most comprehensive development and action plan in the history of NYC. The PlaNYC refers to social sensitive groups in various respects, e.g., to housing, brownfields, air quality, and health, but with little reference to climate change and its impacts. There is a general stress on the decreasing “affordability” of housing for low- and middle-income groups and on mixed income communities. Interesting is the mention of “borough-equity” when it comes to municipal waste. In the chapter on impacts of climate change heat, health and the elderly are mentioned and recommendations for specific communities are given. Other keyword hits are mainly related to development issues but not to climate change impacts, particularly the numerous hits for children. Many planed education programs, school improvements, etc. are mentioned, without the relation to climate, weather impacts, or sensitivity. It was also noted that racial and ethnic diversity is not a reference point in any of the chapters.

The last study in our selection *Responding to Climate Change in New York State – ClimAID Synthesis Report* (NYS ClimAID) is a manifest of social vulnerability and vulnerability analysis. It concurrently underlines the vulnerability of socially sensitive groups and lists vulnerable groups and/or vulnerable places for each chapter. It is an extremely elaborated and socially respective report. Noteworthy, the study provides a map of the distribution of average availability of air-conditioning, for which it is difficult to get data, but points out that many low-income households may be reluctant to use air-conditioning, because of high energy costs. Interestingly, this study is a state report and not commissioned by or prepared for the city.

## Supplementary Table S2 - List of planning documents analyzed in NYC

| Date | Title | Author/ agency/ organization/ publisher | Geographical scale/ scope |
| --- | --- | --- | --- |
| June 2000 | Climate Change and a Global City: An Assessment of the Metropolitan East Coast Region, Assessment Synthesis | U.S. Global Change Research Program & Columbia university research group plus stakeholders | 31 counties of the NYC metro area |
| May 2008 | Climate Change Program Assessment and Action Plan, Report 1 | The NYC Department of Environmental Protection | New York City |
| March 2009 | New York City Natural Hazard Mitigation Plan | NYC Office of Emergency Management | New York City |
| May 2010 | Climate Change and Adaptation in New York City: Building a Risk Management Response | New York City Panel on Climate Change, Mayor’s Office of Long Term Planning and Sustainability, Annals of the New York Academy of Sciences | New York City |
| December 2010 | New York State Sea Level Rise Task Force - Report to the Legislature | Sea Level Rise Task Force | New York State |
| March 2011 | Vision 2020: New York City Comprehensive Waterfront Plan | Department of City Planning, City of New York | New York City |
| April 2011 | PlaNYC Update April 2011 – A greener, greater New York | The City of New York, Office of Long -term Planning and Sustainability | New York City |
| December 2011 | Responding to Climate Change in New York State – ClimAID Synthesis Report | New York State Energy Research and Development Authority | New York State |

## Supplementary Table S3 - Planning practice: Are NYC’s planning strategies socially integrative? (Planning document study)

| Title | Synopsis +  Content Summary | Example quotes with reference to social vulnerability |
| --- | --- | --- |
| Climate Change and a Global City:  An Assessment of the Metropolitan East Coast (MEC) Region,  Assessment Synthesis | The MEC Assessment of Potential Climate  Variability and Change is one of eighteen regional components of the U.S. National  Assessment of the Potential Consequences of Climate Variability and Change for the  Nation. /  The report explicitly mentions the assessment of people (i.e., socio-demographic conditions), place (i.e., physical and ecological systems), and pulse (i.e., decision-making and economic activities) and how these react and respond to climate variability and change. It focused on coasts, wetlands, transportation infrastructure, water supply management, public health, energy, and institutional decision making. The report includes five different climate scenarios and provides a number of different projections for the future climate of the region based on these scenarios. The report proposes three different institutional adaptations to climate change, including the use of climate impact indicators, the creation of an inter-agency climate task force, and a climate awareness program. | p4: “equity of impacts”  p8: “increasing spatial inequity within the region with respect to income levels… high poverty levels correspond with lower access to adequate health care and other social services.”  p8: “many areas, both in urban and suburban, have significant ethnic, black and Hispanic populations. The region also has large populations of elderly and immuno-compromised people."  p9: “several stresses … include … inequity among the region’s residents”  p10: “growing inequity in the MEC region” |
| Climate Change Program Assessment and Action Plan, Report 1 | This report is the NYC’s Department of Environmental Protection’s (DEP) Action Plan and Climate Change Program concentrating on water supply, drainage, and wastewater management services. /  Through this program, DEP assesses the impacts of a warming Earth on New York City's water systems and identifies opportunities for meaningful change. The report concentrates on programs that address New York City's drinking water delivery, storm water management and wastewater treatment systems when assessing four themes: global and regional climate trends, potential climate change impacts on DEP, potential adaptation strategies for DEP, and greenhouse gas emissions and mitigation at DEP. DEP Actions are listed for each topic and a comprehensive Climate Change Action Plan concludes the volume. | No mentioning of social or socio-economic sensitivities. |
| New York City Natural Hazard Mitigation Plan | This Natural Hazard Mitigation Plan outlines the process and steps towards a reduction of the potential impacts of natural hazards on NYC. /  The development of the plan involved various city departments, research organizations, business representatives, community groups, and stakeholder agencies, which developed and laid out the process towards successful hazard mitigation actions. The report details out 5 steps towards successful hazard mitigation from the planning process to the natural hazard risk assessment, the mitigation strategy, the plan adoption, and plan maintenance. The hazard assessment involves a comprehensive analysis of the hazard environment (natural, social and built environment), the population and development trends. Following hazards were investigated: coastal erosion, coastal storms, droughts, earthquakes, extreme temperatures, flooding, windstorms and tornadoes, and winter storms. For each of these hazards a hazard profile as well as a vulnerability assessment was conducted. The plan acknowledges the vulnerability of certain socio-economic groups towards heat waves and maps the geographical distribution of those groups across the NYC area. | p300: “Advocate to expand Weatherization, Referral, and Packaging Program to help low-income seniors… and people with disabilities weatherize their homes against extreme cold and heat events”; “Advocate to expand Home Emergency Assistance Program to include financial assistance to low-income seniors and people with disabilities who require help paying electric bills for air conditioning during extreme heat events”  p143: drought impacts …”increased poverty”  p162: “For both extreme heat and cold, there are geographic variations in vulnerability due to demographic features, such as concentrations of seniors, young children, and individuals living below the poverty line (who are less likely to have adequate heat and air conditioning).”  p167: “New York City also has a large number of individuals who may be susceptible to extreme heat conditions, such as seniors and those living below the poverty line.”  p168: “The following groups are vulnerable or at greater risk to extreme temperatures: People who are homeless, Infants and small children under age five, People age 65 or older, People who are obese, People with medical conditions, People who work outdoors, Women who are pregnant, People who are poor.” |
| Climate Change and Adaptation in New York City: Building a Risk Management Response | This is the report of the NYC Panel on Climate Change (NPCC)–a group of climate impact scientists and legal, insurance, and risk management experts convened by Mayor Bloomberg as part of the City's long-term sustainability plan PlaNYC. /  This volume presents the NPCC’s work regarding the general approach and framing of urban climate change adaptation in NYC (flexible adaptation and mitigation pathways), climate change observations and projections, infrastructure impacts and adaptation challenges, as well as specific tools for adaptation, such as law and regulation, the insurance industry and indicator and monitoring approaches. Due to its nature being developed by scientists and practitioners, the report does not formulate actions. It provides a climate change and impact assessment with a concentration on infrastructure, and suggests possible pathways for adaptation. | p29: “Responding to climate change … takes into account climate change equity”  p37: “Any iterative, or Flexible Adaptation Pathways, process must recognize … equity”  p79: [The London plan includes]… “things such as poor air quality affecting the elderly”  p104: “Worthy projects with marginal funding, such as low-income housing, might not proceed if the financial barriers are too steep.”  p118: “government financial help … might take the form of financial aid to subsidize low-income home owners that require retrofits or other adaptation measures.”  p123: “provide financial aid to subsidize low-income homeowners for retrofits”  p129: “Shifts in …income…might also lead to such Flexible Adaptation Pathways” |
| New York State Sea Level Rise Task Force - Report to the Legislature | The sea level rise task force was charged to prepare a report addressing the impacts of SLR in NYC, including recommendations for an action plan to protect coastal communities and natural resources from rising sea. /  The report outlines the causes and projections of SLR and its potential impacts on various ecosystems (such as tidal wetlands, low‐ to moderate‐energy shorelines, submerged aquatic vegetation, barrier islands, etc,), public works and infrastructure (communications, energy, shoreline protective structures, solid waste, etc.), as well as communities (with relation to health, loss of shelter, disrupted livelihoods, etc.). The report dedicates a whole chapter to climate justice, outlines adaptation champions, and gives recommendation. | p48: “In many cases, communities, especially low‐income communities, have invested considerable time and energy to secure these amenities, and their full value may not be reflected in typical vulnerability assessments. Such losses degrade the quality of life for shoreline communities.”  p49: “Because low‐income communities of color could be disproportionately affected, their participation is critical to adaptation and community resilience planning efforts.”  p51: “Particular effort and funding should be dedicated to ensuring that lower‐income communities and communities of color are adequately involved in planning efforts. The members of such communities often have less discretionary time and resources to devote to participation, and without adequate representation, their needs may be overlooked.”  p74: “relevant stressors such as demographic changes, economic downturns and poverty” |
| Vision 2020: New York City Comprehensive Waterfront Plan | Vision 2020 presents an assessment of the current status of New York City’s waterfront areas and waterways, and also provides an “Action Agenda”, a set of goals and projects to be accomplished by 2020. /  The report (update from 1992) establishes eight broad goals of which one is “Identify and pursue strategies to increase the city’s resilience to climate change and sea level rise”, and offers hundreds of recommendations for the waterfront and waterways for the next decade and beyond. Vision 2020 details achievements made to date on the NYC waterfront, provides a summary of the strategies and projects intended in order to accomplish each of the eight goals on the city level, and lists strategies planed on the neighborhood. The report mainly focuses on improving NYC waterfront and waterways because of the valuable resources they provide. One chapter assesses climate change impacts and potential adaptation/resiliency strategies. | p33: “Funding park maintenance through dedicated funding from nearby developers, property owners, and tenants can potentially create inequities in park upkeep if the outcome is that parks in high-rent neighborhoods receive sufficient funding while parks in lower-income communities do not”  p38: “People of all income levels will need places to live.”  p38: “could provide mixed-income housing”  p38: “provide … units of mixed-income housing”  p42: “jobs for people of diverse income levels”  p42: “Create a waterfront where the city’s needs for new housing and jobs for people of diverse income levels are satisfied” |
| PlaNYC Update April 2011 – A greener, greater New York | First released in 2007, PlaNYC is an effort  to prepare for one million more residents, strengthen  our economy, combat climate change, and enhance the quality of life for all New Yorkers. /  The Plan is a comprehensive planning program that brought together over 25 City agencies to work towards the vision of a greener, greater New York, which should be accomplished through 127 initiatives already outlined in the 2007 Plan. The 2011 report concentrates on 10 themes (of 19 in the 2007 report) plus 1 chapter on cross-cutting topics. One theme is climate change (mitigation and adaptation). Annual progress reports assess the implementation progress of the proposed initiatives. | p20: “we must encourage mixed-income communities that provide a variety of housing choices available to households at a range of incomes.”  p23: “we have implemented targeted affordability programs aimed at low- and middle income New Yorkers through Mayor Bloomberg’s New Housing Marketplace Plan… To accommodate our growing population and improve the affordability of housing for New Yorkers of all incomes, we will propose regulatory changes and other actions to enable the creation of new housing units.”  p24: “The Inclusionary Housing Program permits an increase in the floor area of residential developments in exchange for the provision of low-income housing.”  p136: “path toward “borough equity... by ensuring that City-collected waste from one borough is not sent to another borough”  p162: “air pollution …the burden is borne disproportionately by the city’s oldest and youngest, lower-income, and more vulnerable populations.”  p202: “Hotter temperatures will increase public health risks, particularly for vulnerable populations such as the elderly”  p155: “People who are older, have heart or lung disease, and who are unable to escape the heat are at an increased risk of death during prolonged periods of hot and humid weather.” |
| Responding to Climate Change in New York State – ClimAID Synthesis Report | The Integrated Assessment for Effective Climate Change Adaptation Strategies in New York State was undertaken to provide decision-makers with cutting-edge information on the state's vulnerability to climate change and to facilitate the development of adaptation strategies. /  The assessment was informed by both local experience and scientific knowledge. As part of the introduction it specifies NY states characteristics in terms of climate risks, adaptation, equity and economic issues. The report acknowledges the need to plan for and adapt to climate change impacts in a range of sectors: Water Resources, Coastal Zones, Ecosystems, Agriculture, Energy, Transportation, Telecommunications, and Public Health. Each chapter summarizes particularly vulnerable groups and particularly vulnerable locations in form of a sub-chapter. | p10: “Equity issues emerge because climate change impacts and adaptation policies can worsen existing inequalities and can also create new patterns of winners and losers.”  p12: “Low-income urban neighborhoods, especially those within flood zones, are less able to cope with climate impacts such as heat waves, flooding, and coastal storms.”  p12: “Elderly, disabled, and health-compromised individuals are more vulnerable to climate hazards, including floods and heat waves. • Low-income groups have limited ability to meet higher energy costs, making them more vulnerable to the effects of heat waves. • Those who lack affordable health care are more vulnerable to climate-related illnesses such as asthma. • Those who depend on public transportation to get to work, and lack private cars for evacuating during emergencies, are vulnerable. • Asthma sufferers will be more vulnerable to the decline in air quality during heat waves”.  p17: “The elderly and people with disabilities tend to be more vulnerable to immediate flood hazards due to limited mobility.”  p18: “Lower-income or non-English-speaking populations may be particularly vulnerable to increasing levels of disease-causing agents in the water supply or contaminants in well water as they may be less aware of government programs and warnings and have less access to health care.”  p21: “Low-income populations living in coastal and near-coastal zones will be less able to recover from damages resulting from extreme weather events than will wealthier populations.”; “Racial and ethnic minorities are more vulnerable to extreme events than nonminority populations; African Americans and Latinos represent a significant portion of the people living in the New York City flood zone.”  p23:” Relocating from high-risk coastal areas will put population pressures on some upland communities, potentially increasing property values and putting low-income people at a disadvantage.”  p33: “Elderly, disabled, and health-compromised residents are especially vulnerable to energy outages associated with extreme climate events.”  p34: “Low-income residents living in urban areas, which are already subject to urban heat island effects, may be especially vulnerable to higher energy costs.”; “The effects of heat islands are especially prominent in many lower income neighborhoods, such as Fordham in the Bronx and Crown Heights in Brooklyn. These neighborhoods often have fewer trees on the street and higher building density, both of which contribute to hotter conditions.”  p34: ”Higher poverty areas of New York City, particularly in northern Manhattan, the South Bronx, and parts of Brooklyn, have lower rates of home air conditioning than other areas”  p38: “Low-income and elderly populations, especially in urban areas, are particularly vulnerable to disruption to transportation services, limiting their ability to get to work”  p39: “People with limited mobility and transportation options would be affected the most, including low-income households, the disabled, and the elderly. These populations also may be less likely to access relief from centralized facilities located beyond walking distance.  p41: “Lower-income populations are more likely to drop landline services; this increases their risk during emergency situations, as a result of their more limited communication options.”  p46: “Certain groups—including the elderly, low-income populations, and minorities—are more vulnerable than others to climate-change-related health risks including heat-related illness and death”; “African Americans and Hispanics are particularly vulnerable to decreased air quality because they tend to live in urban centers where they are more exposed to air pollutants”  p46: “Others, including low-income elderly residents—particularly those living alone—may be reluctant to use air conditioning even if they have it due to concerns about energy costs, even during periods of extreme heat.”  p47: “Dampness of households, a key variable for mold growth, is associated with socioeconomic status and could intensify with projected precipitation increases. Mold may contribute to the high rates of hospitalization for asthma among African Americans in cities such as New York” |

Underlined passages refer to climate change impacts on social sensitive groups

## Supplementary Table S4 – Detailed overview of hits per hit category and report

|  | Inequality, equity, justice | (Low-) income | Poverty, poor | Homeless | Elderly/ ies, senior, older | Children, kids, infant | Race, racial, ethnic | Minority/ ies | African(-) American, black, color | Hispanic, Latino | Gender, sex, female, women | Cars | Total # of hits |
| --- | --- | --- | --- | --- | --- | --- | --- | --- | --- | --- | --- | --- | --- |
| Metro EC | 4 | 1 | 1 |  | 1 |  | 1 |  | 1 | 1 |  |  | 10 |
| CC Ass + Action |  |  |  |  |  |  |  |  |  |  |  |  | - |
| NYC Nat Hazards |  | 2 | 5 | 7 | 18 | 8 |  |  |  |  | 2 |  | 42 |
| CC & Adap in NYC | 2 | 4 |  |  | 1 |  |  |  |  |  |  |  | 7 |
| NYS SLR TF | 6 | 3 | 1 |  | 2 | 1 |  |  | 2 |  |  |  | 15 |
| Vision 2020: Waterfr | 1 | 6 |  |  |  | 7 |  |  |  |  |  |  | 14 |
| PlaNYC 2011 | 3 | 12 | 4 |  | 8 | 15 |  |  |  |  |  |  | 42 |
| ClimAID | **3** | **18** | **3** |  | **13** | **4** | **2** | **2** | **3** | **2** | **1** | **1** | **52** |
| Total hits | 19 | 46 | 14 | 7 | 43 | 35 | 3 | 2 | 6 | 3 | 3 | 1 | 182 |

## Supplementary Table S5 - Comparison of Social Vulnerability Indicator when using the additive model with and without weighting and the reductionist model with PCA

| Models | Difference |  |  |
| --- | --- | --- | --- |
| Additive-no weighting & PCA | Absolute change | 138.58 | 491.40 |
|  | Score change | 585 (27%) | 1655 (76%) |
| Additive-weighting & PCA | Absolute change | 95.00 | 317.56 |
|  | Score change | 381 (18%) | 1380 (64%) |

## Supplementary Figures S1

| Score change: **Additive-no weighting & PCA** | 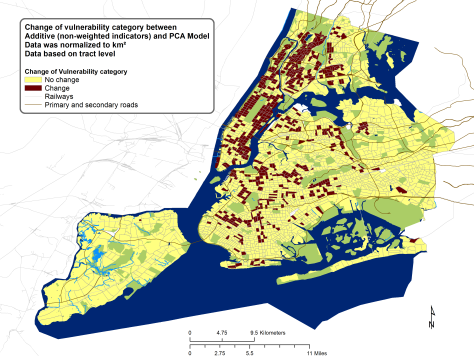 | 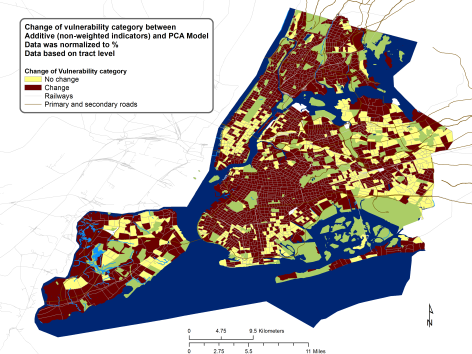 |
| --- | --- | --- |
| Absolute change betw. **Additive-no weighting & PCA (orange shows higher values for PCA)** | 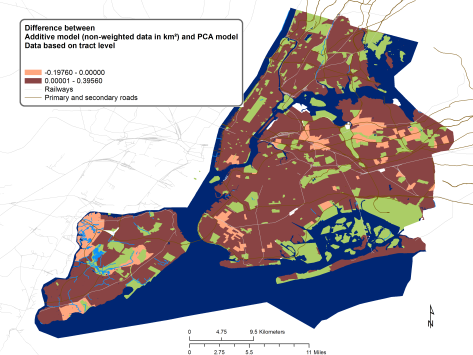 | 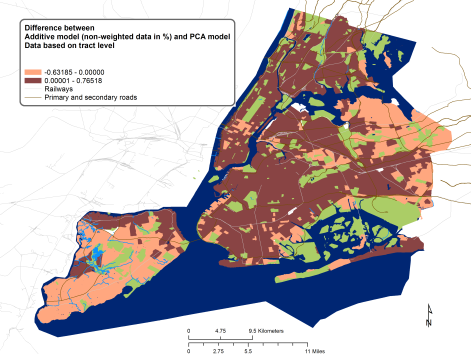 |
| Score change: **Additive-weighting & PCA** | 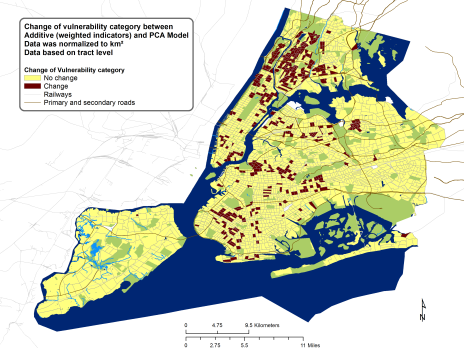 | 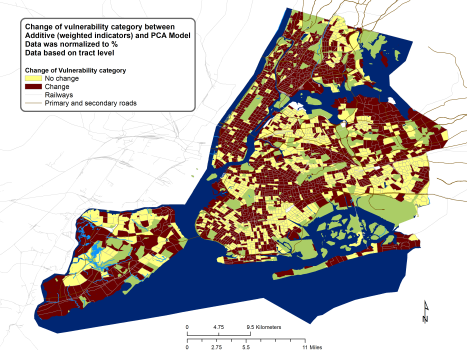 |
| Absolute change betw. **Additive-weighting & PCA (pink shows higher values for PCA)** | 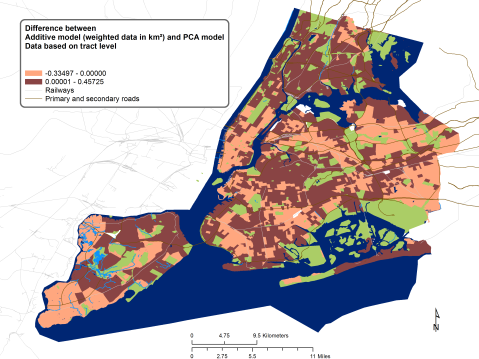 | 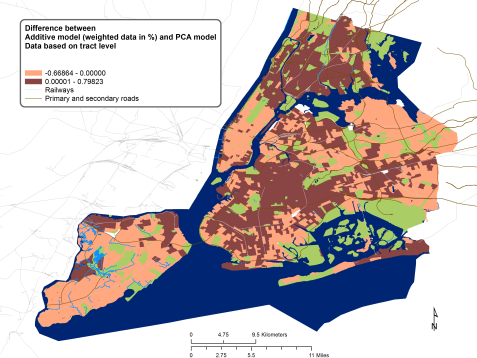 |

## Supplementary Figures S2 – Absolute change between models with and without weighting

|  | Area-based data (per km^2^) |
| --- | --- |
| Absolute change between additive models. **Coral shows higher values for weighted model**. | 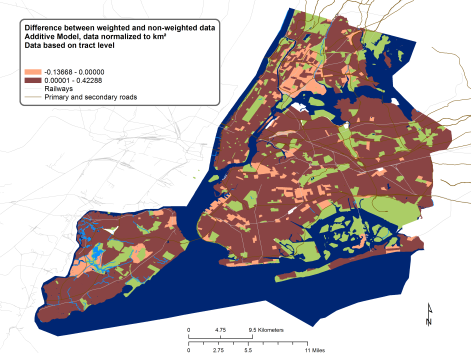 |
|  | Population-based data (%) |
|  | 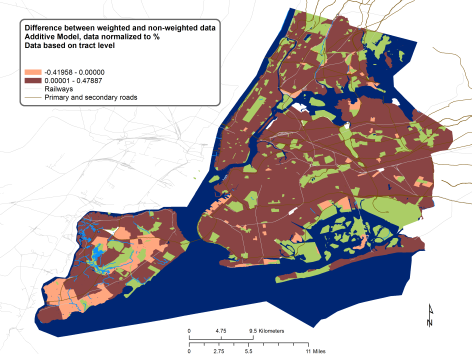 |

Change in social vulnerability indices between pairs of models (additive, with and without weighting) using area and population-based data. Brown depicts tracts where the additive model without weighting has higher scores; coral depicts tracts where the additive model with weighting has higher scores. Non-residential areas are shown in blue (water bodies), green (parks) and white (industrial areas, etc.).

## Supplementary Figures S3 – Histograms and statistics


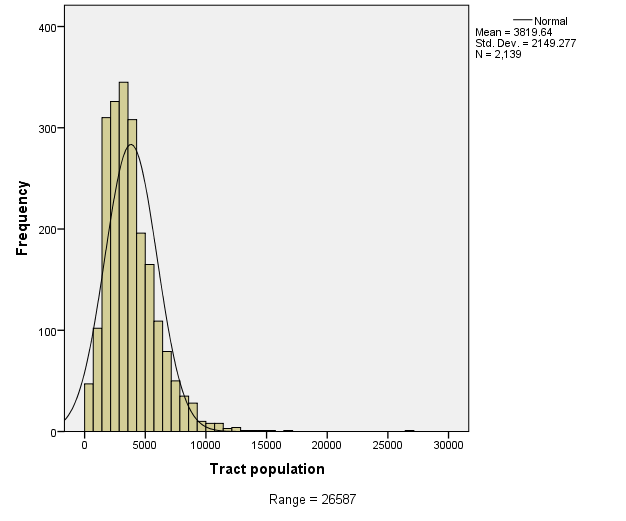


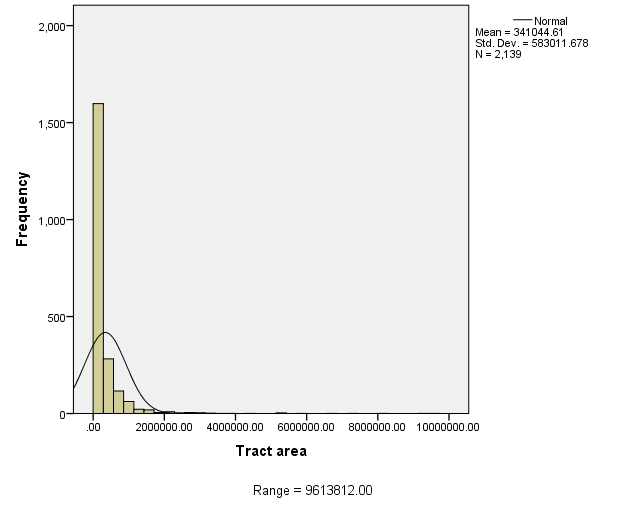


## Supplementary Figures S4 – Absolute change between metrics 1

|  | Absolute change between indices constructed with area-based (per km^2^) and population-based (%) data |
| --- | --- |
| Additive model without weighting | 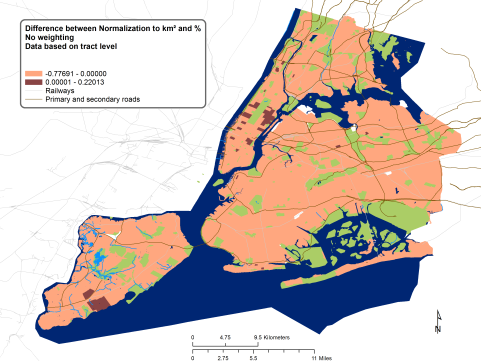 |
| Additive model with weighting | 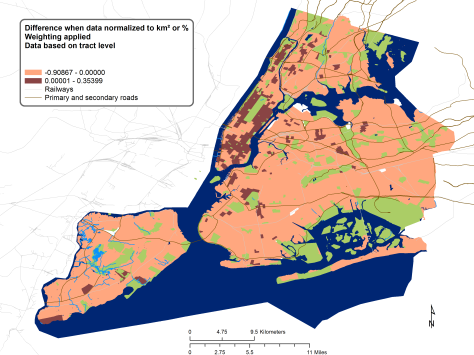 |

Changes between indices constructed with area-based (per km^2^) and population-based (%) data for the additive model, with and without weighting. Dark brown depicts tracts that have a higher vulnerability when using area-based data; coral depicts areas of higher vulnerability using population-based data. Non-residential areas are shown in blue (water bodies), green (parks) and white (industrial areas, etc.).

## Supplementary Figures S5 – Absolute change between metrics 2

|  | Absolute change between indices constructed with area-based (person/ km^2^) and population-based (%) data | |
| --- | --- | --- |
| PCAs | 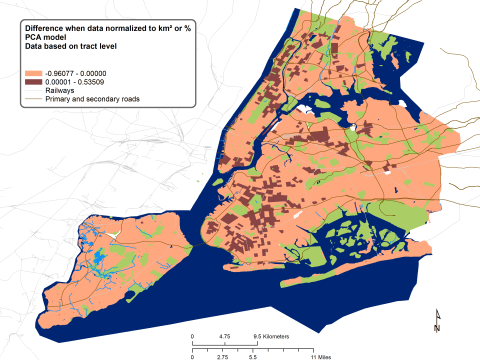 |  |

Changes between indices constructed with area-based (per km^2^) and population-based (%) data for PCA model. Dark brown depicts tracts that have a higher vulnerability when using area-based data; coral depicts areas of higher vulnerability using population-based data. Non-residential areas are shown in blue (water bodies), green (parks) and white (industrial areas, etc.).
